# Craniocervical Junction Morphology and Cerebrospinal Fluid Spaces at the Foramen Magnum in Idiopathic Intracranial Hypertension: Implications for Pathophysiology

**DOI:** 10.3390/jcm15166301

**Published:** 2026-08-14

**Authors:** Recai Engin, Melih Van, Fatih Tomakin, Cem Demirel, Hasan Şener, Muhammed Kırkgeçit, Ahmet Hakan Bayram, Mehlika Berra Pamuk, Bilge Piri Çınar, Ersoy Kocabicak

**Affiliations:** 1 Department of Neurosurgery, Samsun University, Samsun 55000, Türkiye; 2 Department of Neurosurgery, Giresun University, Giresun 28200, Türkiye; 3 Megapoint Hospital, Kahramanmaraş 46100, Türkiye; mkirkgecit46@gmail.com; 4 Department of Neurology, Samsun University, Samsun 55000, Türkiyeberraozberk@gmail.com (M.B.P.);; 5 Department of Neurosurgery, Atlas University, İstanbul 34408, Türkiye

**Keywords:** idiopathic intracranial hypertension, craniocervical junction, foramen magnum, magnetic resonance imaging, cerebrospinal fluid, candidate imaging marker

## Abstract

**Background:** Although numerous magnetic resonance imaging (MRI) findings have been described in idiopathic intracranial hypertension (IIH), the potential contribution of craniocervical junction morphology to disease pathophysiology and diagnosis remains largely unexplored. This study aimed to evaluate foramen magnum morphology and cerebrospinal fluid (CSF) spaces at the craniocervical junction in patients with IIH and to investigate their associations with disease severity and diagnostic performance. **Methods:** In this retrospective case–control study, 70 patients diagnosed with IIH according to the modified Dandy criteria and 70 age- and sex-matched controls were included. Brain MRI examinations were retrospectively reviewed. Optic nerve sheath diameter, optic nerve tortuosity angle, foramen magnum anteroposterior and transverse diameters, foramen magnum area, foramen magnum index, and anterior and posterior subarachnoid CSF spaces were measured. Correlation analyses with lumbar puncture opening pressure and papilledema grade were performed. Independent imaging predictors of IIH were identified using multivariable logistic regression analysis, and diagnostic performance was assessed using receiver operating characteristic (ROC) analysis. **Results:** Compared with controls, patients with IIH demonstrated significant alterations in craniocervical junction morphology, including smaller foramen magnum anteroposterior diameter, foramen magnum area, foramen magnum index, and anterior subarachnoid CSF space, together with increased optic nerve sheath diameter and reduced optic nerve tortuosity angle (all *p* < 0.001). Foramen magnum morphometric parameters and anterior subarachnoid CSF space were significantly associated with both lumbar puncture opening pressure and papilledema grade. Multivariable logistic regression identified optic nerve sheath diameter (OR = 2.143, *p* = 0.002), optic nerve tortuosity angle (OR = 0.925, *p* < 0.001), and foramen magnum index (OR = 0.526 per 0.1-unit increase, *p* = 0.001) as independent imaging predictors of IIH. The combined prediction model demonstrated excellent diagnostic performance (AUC = 0.901), with 81.4% sensitivity and 87.1% specificity. **Conclusions:** Craniocervical junction morphometric features and subarachnoid CSF spaces at the foramen magnum differed between patients with IIH and controls and may provide complementary imaging information. These cross-sectional associations do not establish whether the observed differences are pre-existing anatomical characteristics or secondary changes related to chronically elevated intracranial pressure. Prospective multicenter studies incorporating dynamic CSF-flow imaging are warranted to validate these findings and clarify the temporal relationship between these morphometric features and disease development.

## 1. Introduction

Idiopathic intracranial hypertension (IIH) is a clinical condition characterized by elevated intracranial pressure in the absence of an identifiable intracranial mass lesion or hydrocephalus. It predominantly affects women of reproductive age and is commonly associated with obesity, although its pathophysiology remains incompletely understood [1,2]. Clinically, patients present with headache, visual disturbances, and papilledema, with the potential for irreversible visual loss if left untreated [3].

Over the past decade, increasing attention has been directed toward the role of cerebrospinal fluid (CSF) dynamics and venous outflow abnormalities in the pathogenesis of IIH. Neuroimaging studies have consistently demonstrated findings such as transverse sinus stenosis, optic nerve sheath distension, and empty sella, suggesting a complex interplay between intracranial pressure regulation and venous circulation [4,5,6]. Despite these advances, current models remain insufficient to fully explain the heterogeneity of the disease and the variability in clinical presentation.

The craniocervical junction (CCJ) represents a critical anatomical interface between the intracranial and spinal compartments, serving as a conduit for CSF circulation between these two spaces. The foramen magnum, in particular, plays a pivotal role in maintaining the equilibrium of CSF flow and pressure gradients across the craniospinal axis [7,8]. Alterations in the morphology of this region may influence CSF pulsatility, compliance, and resistance to flow, thereby contributing to intracranial pressure dysregulation. While such mechanisms have been extensively studied in conditions like Chiari malformation (CM), their potential relevance in IIH has not been adequately explored [9].

Previous studies in IIH have primarily focused on intracranial structures and venous abnormalities, while the CCJ has received relatively little attention. However, anatomical variations at the level of the foramen magnum and surrounding subarachnoid CSF spaces may influence CSF exchange between the cranial and spinal compartments, suggesting a potential role beyond a passive anatomical boundary.

In this context, the present study investigates the relationship between CCJ morphology, foramen magnum dimensions, and CSF space in patients with IIH. We further explore whether these anatomical features are associated with disease presence and severity, and hypothesize that alterations at this level may contribute to impaired CSF dynamics and the pathophysiology of IIH. Understanding the potential contribution of the CCJ to IIH could not only refine current pathophysiological models but also open new perspectives for diagnostic evaluation and risk stratification.

## 2. Materials and Methods

### 2.1. Study Design and Participants

In this retrospective study, patients diagnosed with IIH who were followed in the Departments of Neurology and Neurosurgery at our institution between 1 January 2018, and 31 December 2025 were retrospectively reviewed. The study was approved by the Samsun University Non-Interventional Clinical Research Ethics Committee (Decision No. GOKAEK 2026/9/13) and was conducted in accordance with the principles of the Declaration of Helsinki.

The diagnosis of IIH was established according to the modified Dandy criteria based on the combination of clinical presentation, neurological and ophthalmological examinations, neuroimaging findings, and cerebrospinal fluid (CSF) opening pressure measured by lumbar puncture [10]. A total of 70 patients who fulfilled the diagnostic criteria and had complete clinical, radiological, and laboratory data were included in the study.

The control group consisted of age- and sex-matched individuals who presented to the neurosurgery outpatient clinic with headache during the same study period but had no evidence of intracranial pathology on clinical or radiological evaluation. All control subjects had normal neurological examination findings and normal cranial magnetic resonance imaging (MRI) findings.

Only individuals aged 18 years or older were eligible for inclusion. For the IIH group, eligibility required documentation of CSF opening pressure at the time of diagnosis, availability of complete blood count results, and ophthalmological records including papilledema grade and visual field examination. Exclusion criteria for both groups included active infection, autoimmune or chronic inflammatory disease, hematological disorders, a history of malignancy, recent trauma or surgery, systemic corticosteroid or immunosuppressive therapy, and pregnancy.

### 2.2. Clinical Assessment

Demographic and clinical data were retrospectively retrieved from the institutional electronic medical records. Age, sex, and body mass index (BMI) were recorded. Presenting symptoms, including headache, diplopia, vomiting, and tinnitus, were documented. In addition, the use of acetazolamide during the treatment course was recorded for all patients in the IIH group.

All patients with IIH underwent a comprehensive ophthalmological examination at the time of diagnosis. Papilledema was graded according to the Frisén classification. Visual field assessment was performed using automated perimetry, and the mean deviation (MD) value was used for statistical analyses.

### 2.3. MRI Evaluation

As part of the radiological evaluation, cranial MRI examinations of all participants were retrospectively reviewed. Conventional imaging assessment included measurements of the optic nerve tortuosity angle (ONTA) and optic nerve sheath diameter (ONSD). All measurements were obtained on standardized axial MRI sections.

**Figure 1:**
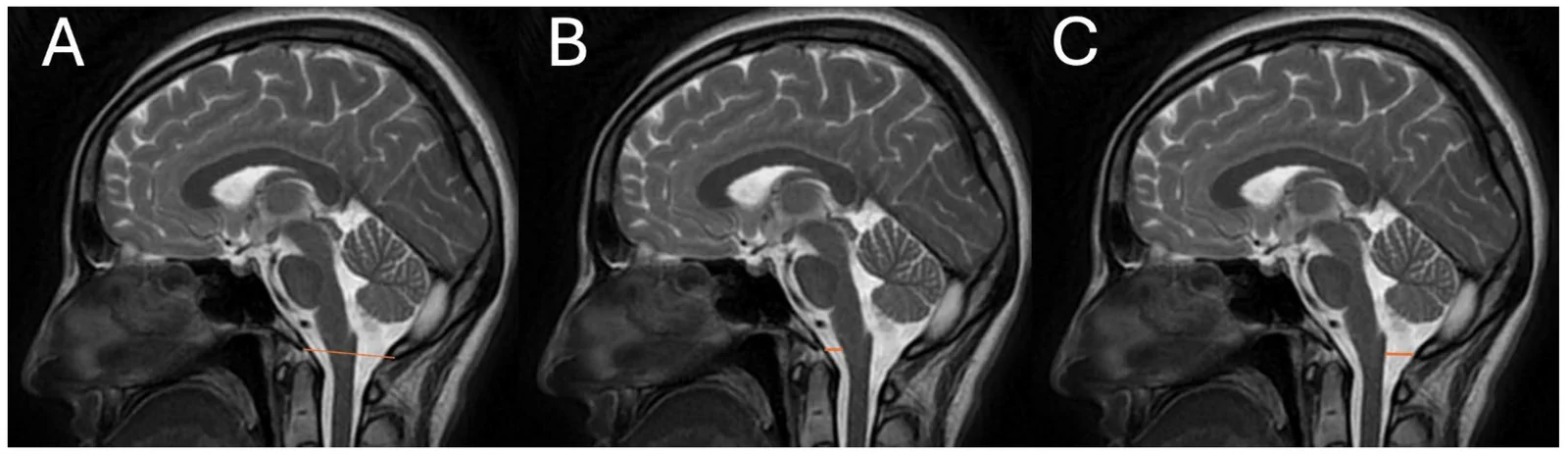
Sagittal T2-weighted magnetic resonance imaging (MRI) illustrating the morphometric measurements performed at the craniocervical junction. (**A**) Measurement of the foramen magnum anteroposterior (AP) diameter, defined as the distance between the basion and the opisthion on the midsagittal image. (**B**) Measurement of the anterior subarachnoid space (aSAS), defined as the shortest distance between the clivus and the anterior surface of the brainstem. (**C**) Measurement of the posterior subarachnoid space (pSAS), defined as the shortest distance between the posterior surface of the brainstem and the posterior margin of the foramen magnum. The orange lines indicate the measured anatomical distances.

**Figure 2:**
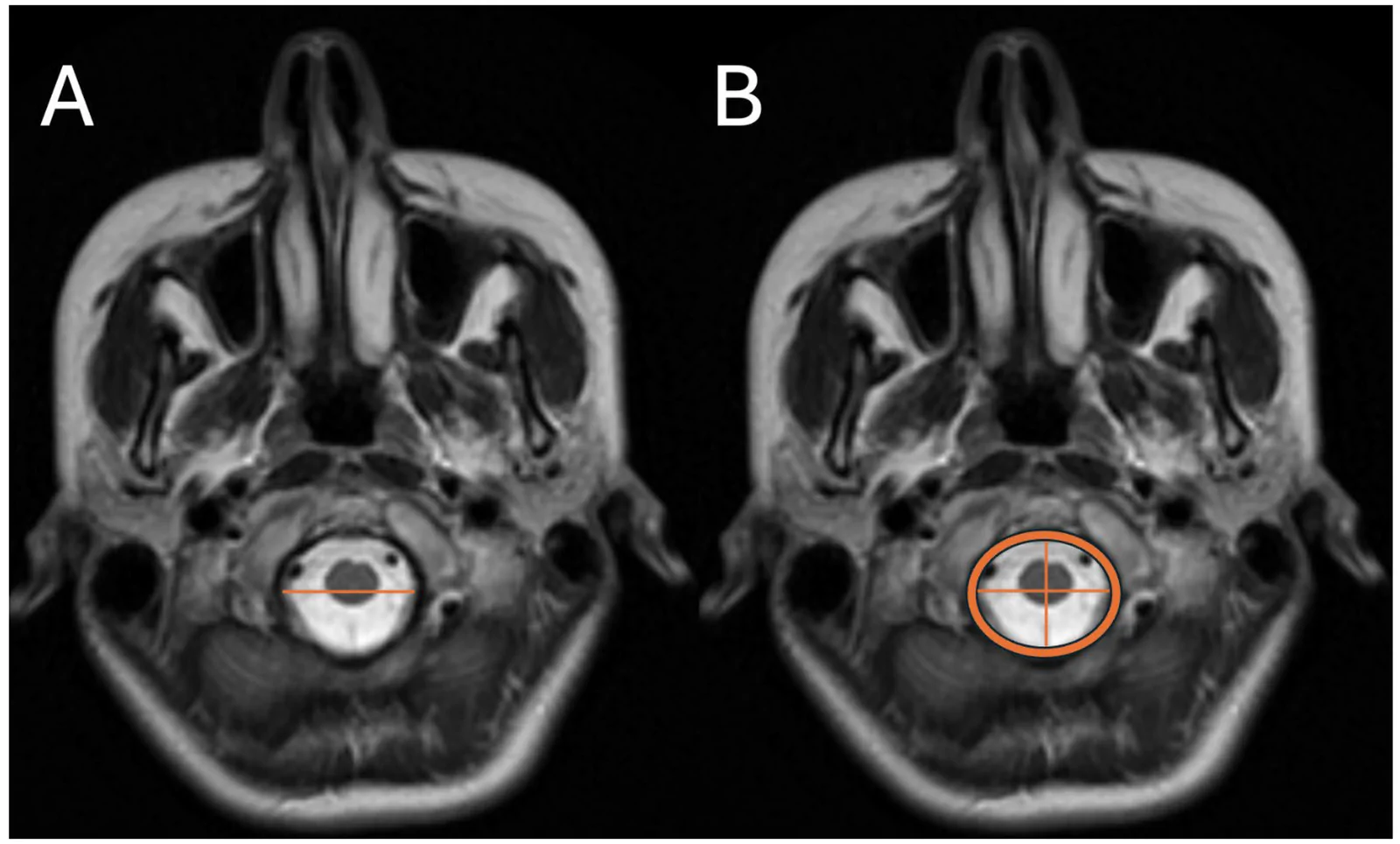
Axial T2-weighted magnetic resonance imaging (MRI) illustrating measurements of the foramen magnum. (**A**) Measurement of the maximum transverse diameter of the foramen magnum. (**B**) Calculation of the foramen magnum area using the ellipse formula based on the anteroposterior and transverse diameters (area = π × AP diameter × transverse diameter/4). The orange ellipse and lines illustrate the measurements used to calculate the foramen magnum area according to the ellipse formula.

In accordance with the primary objective of the study, the CCJ was evaluated in detail. Foramen magnum morphology and CSF spaces at this level were assessed. On midsagittal MRI images, the basion–opisthion distance was measured as the anteroposterior diameter of the foramen magnum (Figure 1). On axial images, the maximum transverse diameter was measured. The foramen magnum area was subsequently calculated using the ellipse formula (π × anteroposterior diameter × transverse diameter/4) (Figure 2). In addition, the foramen magnum index (FMI) was calculated as the ratio of the anteroposterior diameter to the transverse diameter.

To evaluate CSF spaces at the CCJ, the distance between the anterior surface of the brainstem and the clivus on midsagittal images was defined as the anterior subarachnoid space (aSAS), whereas the distance between the posterior surface of the brainstem and the posterior margin of the foramen magnum was defined as the posterior subarachnoid space (pSAS). All measurements were performed using standardized anatomical landmarks. All MRI measurements were performed retrospectively by a single neurosurgeon.

Lumbar puncture was performed in the lateral decubitus position in all patients with IIH, and CSF opening pressure was measured in cmH_2_O using a standard manometric technique.

### 2.4. Statistical Analysis

Statistical analyses were performed using IBM SPSS Statistics for Windows, version 25.0 (IBM Corp., Armonk, NY, USA). The distribution of continuous variables was assessed using the Shapiro–Wilk test. Normally distributed variables are presented as mean ± standard deviation (SD), whereas non-normally distributed variables are presented as median (interquartile range [IQR]). Comparisons between groups were performed using the independent-samples *t*-test or the Mann–Whitney U test, as appropriate. Categorical variables were compared using the chi-square test.

Correlations between CSF opening pressure and radiological parameters were evaluated using Spearman’s rank correlation analysis. Multivariable logistic regression analysis was performed to identify radiological parameters independently associated with IIH. Before model construction, multicollinearity was assessed using the variance inflation factor (VIF) and tolerance values. The diagnostic performance of significant variables was evaluated using receiver operating characteristic (ROC) curve analysis. A two-sided *p* value of <0.05 was considered statistically significant for all analyses. To address the issue of multiple comparisons, the Benjamini–Hochberg (BH) false discovery rate (FDR) procedure was applied separately for each family of tests, including radiological group comparisons and correlation analyses. FDR-adjusted *p* values (q values) are reported alongside unadjusted *p* values where appropriate, and findings that lost statistical significance after FDR correction are explicitly indicated.

Two logistic regression models were constructed. The reduced model included age, sex, BMI, ONSD, and ONTA as predictors. The full model additionally incorporated the FMI, recoded per 0.1-unit increments (FMI × 10) to improve interpretability of the odds ratio. Both models were estimated using maximum likelihood. Model calibration was assessed using the Hosmer–Lemeshow goodness-of-fit test (g = 10 deciles). Discriminative performance was evaluated by receiver operating characteristic (ROC) curve analysis; the area under the curve (AUC) was compared between models using the DeLong method. Internal validation was performed via bootstrap resampling (1000 iterations), yielding the optimism-corrected AUC for each model. The estimated events-per-variable (EPV) ratio was computed to assess model stability. Model fit was additionally summarized using Nagelkerke’s pseudo-R^2^, the Akaike information criterion (AIC), and the Bayesian information criterion (BIC). For the FMI and aSAS, positive predictive value (PPV) and negative predictive value (NPV) at the Youden-index optimal cut-off are reported. The complete logistic regression equation (intercept plus beta coefficients for ONSD, ONTA, and FMI per 0.1 unit) is provided; the predicted probability threshold of ≥0.590 represents the Youden index optimal cut-off derived from the present sample and should not be applied to external populations without prospective validation.

Generative artificial intelligence (ChatGPT, OpenAI) was used solely for English language editing and improving the clarity and readability of the manuscript. The authors take full responsibility for the scientific content of the article, including the study design, data collection, data analysis, interpretation of the results, and the final version of the manuscript.

## 3. Results

### 3.1. Demographic and Clinical Characteristics

A total of 140 individuals were included in the study, comprising 70 patients diagnosed with IIH according to the modified Dandy criteria and 70 age- and sex-matched controls. There were no significant differences between the IIH and control groups with respect to age or sex distribution (*p* = 0.641 and *p* = 1.000, respectively). However, BMI was significantly higher in the IIH group (*p* = 0.003). The most common presenting symptom among patients with IIH was headache (87.1%), followed by diplopia (71.4%), visual field defects (62.9%), nausea/vomiting (27.1%), and tinnitus (17.1%) (Table 1).

### 3.2. Comparison of Radiological Parameters Between the IIH and Control Groups

Comparison of radiological parameters between the IIH and control groups demonstrated significant differences in ONSD, ONTA, foramen magnum anteroposterior diameter, foramen magnum area, FMI, and aSAS (all *p* < 0.001). Compared with the control group, patients with IIH had a significantly greater ONSD, whereas the ONTA, foramen magnum anteroposterior diameter, foramen magnum area, FMI, and aSAS were significantly smaller. In contrast, no significant differences were observed between the groups with respect to the transverse diameter of the foramen magnum or the pSAS (*p* = 0.889 and *p* = 0.896, respectively) (Table 2).

### 3.3. Associations Between CSF Opening Pressure, Papilledema Grade, and Radiological Parameters

In the IIH group, Spearman correlation analysis demonstrated moderate negative correlations between CSF opening pressure and the foramen magnum anteroposterior diameter (ρ = −0.520, *p* < 0.001), transverse diameter (ρ = −0.421, *p* < 0.001), foramen magnum area (ρ = −0.571, *p* < 0.001), and aSAS (ρ = −0.504, *p* < 0.001). In contrast, no significant correlations were observed between CSF opening pressure and ONSD, ONTA, FMI, or pSAS (Table 3).

Papilledema grade was significantly negatively correlated with the foramen magnum anteroposterior diameter (ρ = −0.741, *p* < 0.001), transverse diameter (ρ = −0.643, *p* < 0.001), foramen magnum area (ρ = −0.800, *p* < 0.001), FMI (ρ = −0.319, *p* = 0.007), aSAS (ρ = −0.721, *p* < 0.001), and pSAS (ρ = −0.268, *p* = 0.025). In addition, ONSD (ρ = −0.499, *p* < 0.001) and ONTA (ρ = −0.397, *p* < 0.001) also showed significant negative correlations with papilledema grade (Table 4).

### 3.4. Independent Radiological Predictors of IIH

After adjustment for age, sex, and BMI, two nested logistic regression models were constructed. The reduced model (ONSD + ONTA + confounders) yielded an AUC of 0.866 (bootstrap-optimism-corrected AUC = 0.845; Nagelkerke R^2^ = 0.515; AIC = 137.7; Hosmer–Lemeshow χ^2^ = 10.327, df = 8, *p* = 0.243). The full model additionally incorporating the FMI (recoded per 0.1-unit increment) yielded an AUC of 0.901 (bootstrap-corrected AUC = 0.879; Nagelkerke R^2^ = 0.589; AIC = 126.5; BIC = 147.1; Hosmer–Lemeshow χ^2^ = 4.836, df = 8, *p* = 0.775). The DeLong test comparing the two models found that the difference not statistically significant (z = 0.857, *p* = 0.391). The number of events per variable for the full model (EPV) was 11.7 (70 events, 6 parameters). In the full model (Table 5), ONSD (β = 0.762, OR = 2.143, 95% CI: 1.338–3.432; *p* = 0.002), ONTA (β = −0.078, OR = 0.925, 95% CI: 0.895–0.955; *p* < 0.001), and FMI per 0.1 unit (β = −0.643, OR = 0.526, 95% CI: 0.362–0.762; *p* = 0.001) were identified as independent radiological predictors of IIH. The aSAS approached but did not reach statistical significance (OR = 0.692, 95% CI: 0.471–1.016; *p* = 0.060) (Table 5). The logistic regression equation is: log-odds (IIH) = 14.972 + 0.762 × ONSD − 0.078 × ONTA − 0.643 × FMI_0_._1_. Good model calibration was confirmed by the Hosmer–Lemeshow goodness-of-fit test (χ^2^ = 4.836, df = 8, *p* = 0.775). The predicted probability threshold of ≥0.590 applied in Table 6 is the Youden index optimal cut-off for this sample; external validation is required before applying this threshold clinically.

### 3.5. Diagnostic Performance of Radiological Parameters

**Figure 3:**
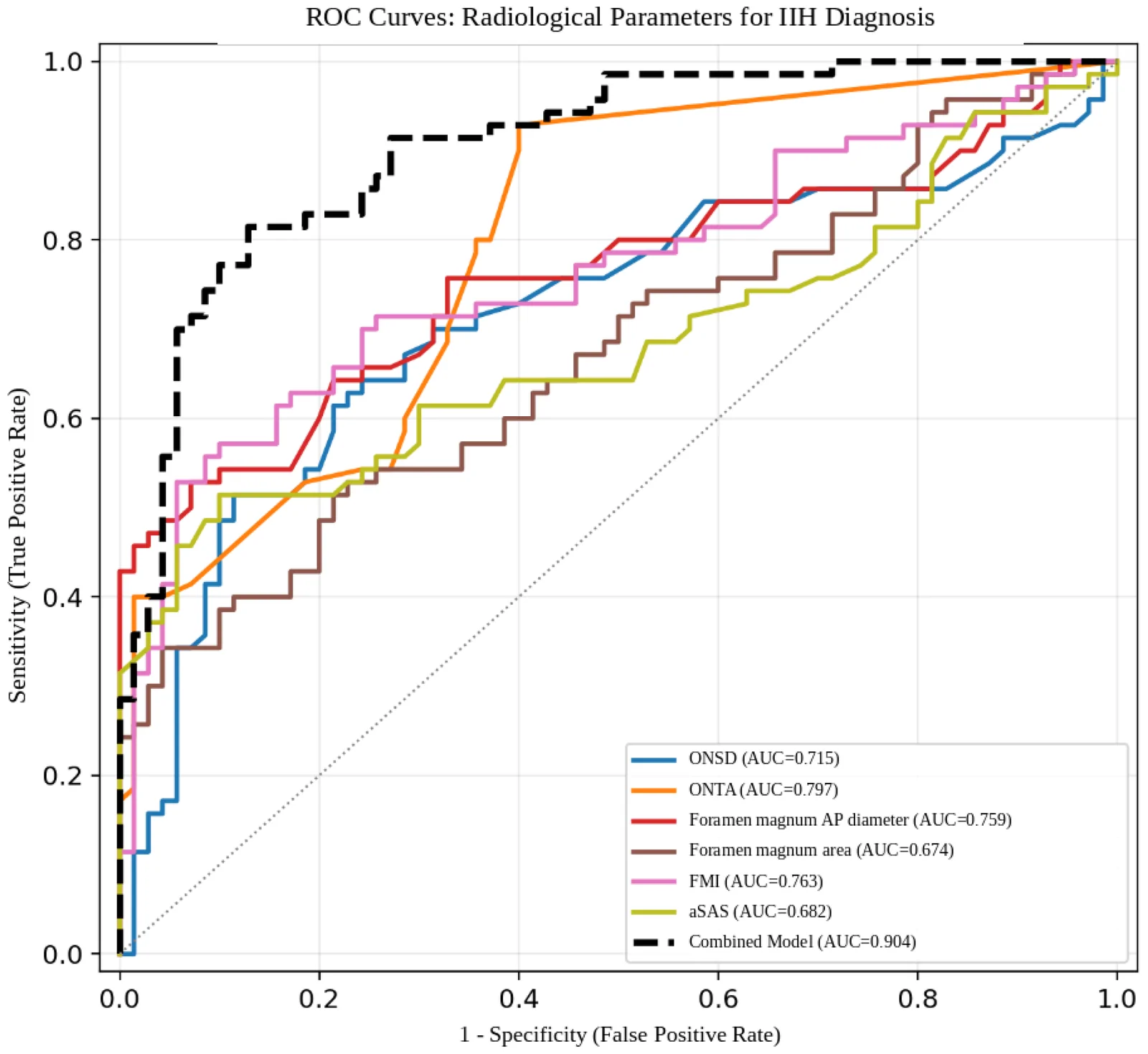
Receiver operating characteristic (ROC) curves for the significant imaging parameters and the combined multivariable logistic regression model for the diagnosis of idiopathic intracranial hypertension. The combined model demonstrated the highest diagnostic performance (AUC = 0.901).

The diagnostic performance of radiological parameters that differed significantly between the IIH and control groups was evaluated using receiver operating characteristic (ROC) curve analysis. Among the individual radiological parameters, the ONTA demonstrated the highest discriminatory performance (AUC = 0.797, 95% CI: 0.723–0.871). This was followed by the FMI (AUC = 0.763) and the foramen magnum anteroposterior diameter (AUC = 0.759). The combined multivariable logistic regression model achieved the highest diagnostic performance (AUC = 0.904, 95% CI: 0.852–0.956), with a sensitivity of 81.4% and a specificity of 87.1% for the diagnosis of IIH (Table 6, Figure 3).

## 4. Discussion

Although the pathophysiology of IIH has not been fully elucidated, several mechanisms, including obesity, disturbances in CSF production and absorption, and venous sinus stenosis, are thought to contribute to disease development [2,11,12,13]. However, the potential role of the CCJ, particularly the anatomical characteristics of the foramen magnum, has received little attention. In the present study, the foramen magnum anteroposterior diameter, foramen magnum area, and aSAS were all significantly smaller in patients with IIH than in controls (all *p* < 0.001). Moreover, these morphometric alterations were associated not only with the presence of IIH but also with CSF opening pressure, papilledema severity, and diagnostic performance. Collectively, these findings suggest that the CCJ may represent a functionally relevant anatomical region that influences CSF dynamics, rather than merely serving as a passive transition zone between the cranial and spinal compartments.

Neuroimaging findings have long been used as diagnostic adjuncts in patients with IIH. Imaging features such as optic nerve sheath enlargement, optic nerve tortuosity, posterior globe flattening, empty sella, and transverse venous sinus stenosis have been well described and reported with varying diagnostic sensitivities and specificities [4,6,14,15,16,17,18]. However, most previous studies have focused on the intracranial compartment, the orbit, and, in particular, venous sinus stenosis. Similarly, Hoffmann et al. characterized morphometric and volumetric MRI changes in patients with IIH but did not extend their evaluation to the CCJ [19]. A distinctive aspect of our study is that it expands the radiological assessment of IIH to the CCJ and demonstrates that foramen magnum morphology may provide complementary diagnostic information beyond established orbital imaging markers. Accordingly, our findings suggest that measurements such as the FMI and the aSAS could be incorporated as complementary parameters into the current MRI-based evaluation of IIH.

The foramen magnum represents the principal anatomical interface between the intracranial and spinal CSF compartments and plays a pivotal role in craniospinal CSF dynamics. Phase-contrast MRI studies have demonstrated that CSF flow and pressure pulsations are continuously regulated across this region throughout the cardiac cycle [7,8]. Likewise, it has long been recognized that anatomical narrowing at the level of the foramen magnum disrupts CSF flow and constitutes a key pathophysiological mechanism in CM [9]. More recently, an editorial by Staartjes et al. emphasized the growing importance of cerebrospinal fluid flow imaging in Chiari malformation, further underscoring the critical role of CSF dynamics across the foramen magnum [20]. In contrast, previous studies on IIH focused predominantly on the venous sinus system and conventional intracranial imaging findings, whereas the potential contribution of the CCJ has remained largely unexplored [2,4,17,21]. In the present study, the significantly smaller foramen magnum anteroposterior diameter, foramen magnum area, and aSAS observed in patients with IIH suggest that anatomical variations at the CCJ may influence craniospinal CSF dynamics. However, our findings do not imply that these anatomical characteristics are causative factors in IIH; rather, they may represent structural features that either contribute to disease susceptibility or develop secondary to the disease process.

The pathophysiological mechanisms of Chiari malformation and IIH are fundamentally different, and the present findings should not be interpreted as evidence of a shared mechanism. Chiari malformation is cited only to illustrate the physiological relevance of the foramen magnum as an interface for craniospinal CSF flow. In the absence of phase-contrast MRI or direct compliance measurements, our data cannot establish whether the observed reduction in aSAS alters CSF flow or craniospinal compliance. This possibility should therefore be regarded as a hypothesis requiring dedicated physiological investigation.

In our cohort, the ONSD was significantly greater in patients with IIH than in controls (median, 4.86 mm vs. 3.90 mm; *p* < 0.001), whereas the ONTA was significantly smaller (median, 150° vs. 180°; *p* < 0.001). Moreover, both parameters remained independently associated with IIH in the multivariable logistic regression analysis. These findings are in agreement with previous studies demonstrating that increased CSF pressure within the perioptic subarachnoid space leads to optic nerve sheath distension and alterations in the normal course of the optic nerve [6,14,15,16,17,18]. Recent reviews have likewise emphasized that optic nerve sheath enlargement and optic nerve tortuosity are among the most reliable MRI markers of IIH, although their diagnostic accuracy is limited when used individually [4,17,18]. In our study, the combined multivariable model achieved superior diagnostic performance (AUC = 0.901) compared with each individual imaging parameter, supporting the concept that integrating complementary MRI markers may improve the diagnostic accuracy of IIH.

One of the most noteworthy findings of the present study is that foramen magnum morphology and the aSAS were associated not only with the presence of IIH but also with CSF opening pressure and papilledema severity. Specifically, smaller foramen magnum anteroposterior diameter, foramen magnum area, and aSAS were associated with higher CSF opening pressure. Similarly, reductions in these parameters were correlated with greater papilledema severity. Notably, the foramen magnum area showed strong negative correlations with both CSF opening pressure (ρ = −0.571, *p* < 0.001) and papilledema severity (ρ = −0.800, *p* < 0.001), suggesting that CCJ morphology may be related not only to anatomical variation but also to disease severity. Nevertheless, because of the cross-sectional design of our study, these associations should not be interpreted as evidence of a causal relationship. Future prospective studies incorporating dynamic CSF flow assessment may further clarify the role of the CCJ in the pathophysiology and progression of IIH.

Notably, neither ONSD nor FMI was significantly correlated with CSF opening pressure within the IIH group. This finding indicates that their ability to distinguish patients with IIH from controls does not necessarily reflect a direct relationship with the magnitude of CSF opening pressure. The correlation and logistic regression analyses addressed different outcomes: disease severity within the IIH cohort and the presence of IIH in the overall study population, respectively.

In the present study, multivariable logistic regression analysis identified ONSD (OR = 2.143, *p* < 0.001), ONTA (OR = 0.925, *p* < 0.001), and the FMI (OR = 0.526 per 0.1-unit increase, *p* = 0.001) as independent imaging markers associated with IIH. Furthermore, the combined model incorporating these parameters demonstrated superior diagnostic performance compared with any individual imaging marker, suggesting that a multiparametric MRI approach may improve the diagnostic evaluation of IIH. Current guidelines and recent reviews emphasize that MRI findings alone are insufficient to establish a diagnosis of IIH but substantially increase diagnostic confidence when interpreted in conjunction with clinical and ophthalmological findings [1,4,17,18,22]. Our findings suggest that incorporating CCJ morphometry into the current MRI assessment may provide complementary diagnostic information, particularly in patients with equivocal clinical presentations. Notably, the combined model based on ONSD, ONTA, and the FMI achieved an AUC of 0.904, outperforming all individual imaging parameters. Growing interest has recently focused on the identification of both imaging and biological biomarkers to improve the diagnosis, monitoring, and understanding of IIH [23,24]. In this context, the CCJ morphometric measurements identified in our study may represent promising candidate imaging markers that warrant further validation. Nevertheless, before these parameters can be incorporated into routine clinical practice, their diagnostic utility should be confirmed in large, prospective, multicenter studies.

The observed morphometric differences may represent pre-existing anatomical variation; however, they could also reflect secondary effects of chronically elevated intracranial pressure or altered intracranial tissue and CSF relationships. The present study cannot distinguish between these possibilities. Longitudinal studies incorporating dynamic CSF-flow imaging will be necessary to clarify the temporal relationship between these morphometric findings and disease development.

The association between FMI and IIH should be interpreted cautiously. Because FMI is a ratio with a narrow observed range, the original odds ratio expressed per one-unit increase appeared numerically extreme. Rescaling FMI per 0.1-unit change (OR = 0.526, 95% CI: 0.362–0.762; *p* = 0.001) provided a more clinically interpretable effect estimate. Furthermore, internal validation and comparison of reduced and full models confirmed model stability and the incremental contribution of FMI: the reduced model (ONSD + ONTA + confounders) achieved an AUC of 0.866 (bootstrap-corrected AUC = 0.845), whereas the full model (ONSD + ONTA + FMI + confounders) achieved an AUC of 0.901 (bootstrap-corrected AUC = 0.879; ΔAIC = 11.2; ΔBIC = 8.2), with substantially improved Hosmer–Lemeshow calibration (*p* = 0.775 vs. *p* = 0.243).

At present, FMI and aSAS should not be used as stand-alone diagnostic criteria or as substitutes for established clinical, ophthalmological, and radiological evaluation. Their potential clinical value lies in providing additional quantitative information when routine MRI is already being reviewed, particularly in patients with otherwise equivocal imaging findings. FMI can be calculated from standard midsagittal and axial MRI measurements without additional imaging sequences, while aSAS can be obtained from a routine midsagittal image. Nevertheless, the proposed thresholds and combined probability model require external validation before any clinical decision algorithm can be recommended.

Obesity may influence the anatomy of the craniocervical junction through increased epidural fat deposition or surrounding soft tissues, potentially reducing measurable subarachnoid CSF spaces independently of IIH-related intracranial pressure abnormalities. Although BMI was included as a covariate in the multivariable model, residual confounding related to obesity cannot be completely excluded. Future studies including BMI-matched control groups and direct assessment of epidural fat may help clarify this potential confounding effect.

The present study cannot determine whether the observed reductions in foramen magnum dimensions and anterior subarachnoid CSF space represent pre-existing anatomical characteristics predisposing individuals to IIH or secondary structural changes resulting from chronically elevated intracranial pressure. Both explanations remain plausible, and longitudinal studies incorporating dynamic CSF-flow imaging will be necessary to clarify the temporal relationship between these morphometric findings and disease development.

Although the combined model demonstrated excellent discrimination within the present cohort, external validation in independent populations is required before routine clinical implementation can be recommended. Furthermore, the model should currently be regarded as a complementary research tool rather than a stand-alone diagnostic instrument. The included morphometric parameters can be obtained from routine MRI without additional imaging sequences; however, standardized measurement protocols and external validation are essential before widespread clinical use. From a practical perspective, these measurements may be particularly useful in patients with equivocal imaging findings or when conventional MRI features are inconclusive.

Beyond its potential influence on bulk CSF movement, the craniocervical junction may also be relevant to recently proposed glymphatic and meningeal lymphatic pathways involved in cerebrospinal fluid circulation and waste clearance [25,26]. Although the present study did not directly evaluate glymphatic function, alterations in craniocervical junction morphology could theoretically influence CSF exchange pathways. These possibilities remain speculative and require dedicated physiological investigations using advanced imaging techniques.

One of the major strengths of this study is that, to the best of our knowledge, it is the first to systematically evaluate both foramen magnum morphology and the subarachnoid CSF spaces at the CCJ in patients with IIH. Whereas previous studies have primarily focused on established imaging findings, such as those concerning the optic nerve, sella turcica, and venous sinus system, our study provides a comprehensive assessment of foramen magnum morphology together with the subarachnoid CSF spaces at this level [4,15,17,18]. Furthermore, the inclusion of both patients and controls, the correlation analyses with clinically relevant measures including CSF opening pressure and papilledema severity, the identification of independent imaging markers using multivariable logistic regression, and the evaluation of diagnostic performance by ROC curve analysis collectively enhance the methodological robustness of our study.

## 5. Limitations

This study has several limitations. First, because of its retrospective, single-center design, the possibility of selection bias cannot be completely excluded. Second, the radiological assessment was based on morphological measurements, and CSF flow dynamics could not be evaluated directly using techniques such as phase-contrast MRI. Consequently, the underlying physiological mechanism linking CCJ morphology to CSF circulation could not be elucidated in the present study. Future studies incorporating larger, multicenter, prospective cohorts together with advanced imaging techniques capable of assessing dynamic CSF flow are warranted to validate our findings and further clarify the role of the CCJ in IIH.

Although all morphometric measurements were performed by a single board-certified neurosurgeon experienced in craniospinal anatomy using predefined anatomical landmarks and a standardized measurement protocol, formal assessment of interobserver and intraobserver reliability was not performed. Future prospective studies involving multiple independent observers are warranted to further evaluate measurement reproducibility.

The control group consisted of individuals presenting with headache who had normal neurological examinations and normal cranial MRI findings rather than asymptomatic healthy volunteers. Therefore, subtle physiological alterations associated with primary headache disorders cannot be completely excluded and may have influenced the observed morphometric findings.

## 6. Conclusions

This study demonstrates that, in addition to established orbital MRI findings, CCJ morphology and the subarachnoid CSF spaces at the level of the foramen magnum are associated with IIH. The observed associations support further investigation of CCJ morphometry as a complementary imaging approach in IIH. However, the cross-sectional design does not permit conclusions regarding causality, temporal sequence, or underlying physiological mechanisms. Longitudinal studies incorporating dynamic CSF-flow imaging are required to determine whether these features predispose to IIH or arise secondary to the disease process.

## Figures and Tables

**Table 1:** Demographic and clinical characteristics of the study population.

Variable	IIH (*n* = 70)	Controls (*n* = 70)	*p*-Value
Age (years)	39.04 ± 12.46	38.17 ± 9.38	0.641
Sex			
Female, n (%)	57 (81.4%)	57 (81.4%)	1.000
Male, n (%)	13 (18.6%)	13 (18.6%)	
BMI (kg/m^2^)	31.19 (27.90–34.55)	29.10 (24.32–31.20)	0.003
Presenting symptoms			
Headache, n (%)	61 (87.1%)	-	
Diplopia, n (%)	50 (71.4%)	-	
Visual field defects, n (%)	44 (62.9%)	-	
Nausea/vomiting, n (%)	19 (27.1%)	-	
Tinnitus, n (%)	12 (17.1%)	-	

Note: Data are presented as mean ± standard deviation, median (interquartile range), or number (%), as appropriate. Continuous variables were compared using the independent-samples *t*-test or the Mann–Whitney U test, and categorical variables were compared using the chi-square test. Presenting symptoms were only evaluated in the IIH group.

**Table 2:** Comparison of radiological parameters between patients with idiopathic intracranial hypertension and healthy controls.

Variable	IIH (*n* = 70)	Controls (*n* = 70)	*p* Value	q Value †
ONSD (mm)	4.86 (4.00–5.80)	3.90 (3.60–4.32)	<0.001	<0.001
ONTA (°)	150.00 (135.00–165.00)	180.00 (156.00–180.00)	<0.001	<0.001
FM anteroposterior diam. (mm)	38.05 (34.15–40.62)	41.65 (39.73–43.26)	<0.001	<0.001
FM transverse diam. (mm)	29.05 ± 2.32	29.10 ± 2.37	0.889	0.896
FM area (mm^2^)	864.09 (738.38–973.56)	957.63 (882.81–1018.38)	<0.001	<0.001
FMI	1.24 (1.20–1.41)	1.42 (1.37–1.52)	<0.001	<0.001
aSAS (mm)	6.25 (4.91–7.88)	7.46 (6.72–8.07)	<0.001	<0.001
pSAS (mm)	12.80 (10.60–14.20)	12.65 (10.80–14.53)	0.896	0.896

Note: Data are presented as mean ± standard deviation or median (interquartile range [IQR]), as appropriate. Normally distributed variables were compared using the independent-samples *t*-test, whereas non-normally distributed variables were compared using the Mann–Whitney U test. FDR-adjusted q values (Benjamini–Hochberg method) are presented alongside raw *p* values. All six a priori significant parameters retained significance after FDR correction (q < 0.05). Foramen magnum transverse diameter (raw *p* = 0.889, q = 0.896) and pSAS (raw *p* = 0.896, q = 0.896) were not significant before or after correction. † q value: Benjamini–Hochberg false discovery rate (FDR)-adjusted *p* value.

**Table 3:** Correlation analysis between CSF opening pressure and radiological parameters in patients with IIH.

Radiological Parameter	Spearman’s ρ	*p* Value	q Value †
ONSD (mm)	−0.148	0.223	0.254 ‡
ONTA (°)	−0.162	0.179	0.239 ‡
Foramen magnum anteroposterior diam. (mm)	−0.520	<0.001	<0.001
Foramen magnum transverse diam. (mm)	−0.421	<0.001	<0.001
FM area (mm^2^)	−0.571	<0.001	<0.001
FMI	−0.228	0.058	0.093 ‡
aSAS (mm)	−0.504	<0.001	<0.001
pSAS (mm)	−0.063	0.602	0.602 ‡

Note: Correlations were assessed using Spearman’s rank correlation analysis. FDR-adjusted q values (Benjamini–Hochberg method) are presented alongside raw *p* values. FM anteroposterior diameter (raw *p* < 0.001, q < 0.001), FM transverse diameter (raw *p* < 0.001, q = 0.001), FM area (raw *p* < 0.001, q < 0.001), and aSAS (raw *p* < 0.001, q < 0.001) remained significant after FDR correction. ONSD (q = 0.254), ONTA (q = 0.239), FMI (q = 0.093), and pSAS (q = 0.602) did not reach significance after correction. † q value: Benjamini–Hochberg false discovery rate (FDR)-adjusted *p* value; q < 0.05 indicates statistical significance after correction for multiple comparisons. ‡ Not significant after FDR correction (q ≥ 0.05).

**Table 4:** Correlation analysis between papilledema grade and radiological parameters in patients with IIH.

Radiological Parameter	Spearman’s ρ	*p* Value	q Value †
ONSD (mm)	−0.499	<0.001	<0.001
ONTA (°)	−0.397	<0.001	<0.001
Foramen magnum anteroposterior diam. (mm)	−0.741	<0.001	<0.001
Foramen magnum transverse diam. (mm)	−0.643	<0.001	<0.001
FM area (mm^2^)	−0.800	<0.001	<0.001
FMI	−0.319	0.007	0.008
aSAS (mm)	−0.721	<0.001	<0.001
pSAS (mm)	−0.268	0.025	0.025

Note: Correlations were assessed using Spearman’s rank correlation analysis. FDR-adjusted q values (Benjamini–Hochberg method) are presented alongside raw *p* values. All eight correlations remained statistically significant after FDR correction (all q < 0.05): ONSD (q < 0.001), ONTA (q = 0.001), FM anteroposterior diameter (q < 0.001), FM transverse diameter (q < 0.001), FM area (q < 0.001), FMI (q = 0.008), aSAS (q < 0.001), and pSAS (q = 0.025). † q value: Benjamini–Hochberg false discovery rate (FDR)-adjusted *p* value; q < 0.05 indicates statistical significance after correction for multiple comparisons.

**Table 5:** Multivariable logistic regression analysis of independent radiological predictors associated with IIH.

Variable	β	SE	Wald χ^2^	OR (95% CI)	*p*-Value	
Constant (Intercept)	14.972	—	—	—	—	
Age	0.028	0.023	1.521	1.028 (0.984–1.076)	0.217	
Sex (male)	0.163	0.625	0.068	1.177 (0.346–4.005)	0.794	
BMI	0.064	0.055	1.346	1.066 (0.957–1.186)	0.246	
ONSD	0.762	0.240	10.051	2.143 (1.338–3.432)	0.002	**
ONTA	−0.078	0.017	22.518	0.925 (0.895–0.955)	<0.001	***
FMI (per 0.1 unit)	−0.643	0.190	11.504	0.526 (0.362–0.762)	0.001	***
aSAS	−0.369	0.198	3.469	0.692 (0.471–1.016)	0.060	

Note: The multivariable logistic regression model was adjusted for age, sex, and BMI. Multicollinearity was assessed before model construction; because of substantial collinearity (VIF > 100), the foramen magnum anteroposterior diameter and foramen magnum area were excluded, and only the FMI was retained. The FMI was rescaled per 0.1-unit increment to improve interpretability of the odds ratio. The table above presents the full model (ONSD + ONTA + FMI; age, sex, BMI as confounders). The logistic regression equation for the three imaging predictors (after adjusting for confounders) is: log-odds (IIH) = 14.972 + 0.762 × ONSD − 0.078 × ONTA − 0.643 × FMI(per 0.1 unit). Model performance: Nagelkerke R^2^ = 0.589; AIC = 126.5; BIC = 147.1; Hosmer–Lemeshow χ^2^ = 4.836 (df = 8, *p* = 0.775); bootstrap-optimism-corrected AUC = 0.879 (1000 iterations). Reduced model (ONSD + ONTA only): AUC = 0.866, corrected AUC = 0.845; DeLong test vs. full model: z = 0.857, *p* = 0.391. Events per variable (EPV) = 11.7. The predicted probability cut-off of ≥0.590 (Table 6) is the Youden index optimal threshold for this sample only and requires prospective external validation before clinical use. Model calibration was evaluated using the Hosmer–Lemeshow goodness-of-fit test. ** *p* < 0.01. *** *p* < 0.001.

**Table 6:** ROC analysis of significant radiological parameters for the diagnosis of IIH.

Parameter	AUC (95% CI)	Optimal Cut-Off	Sensitivity (%)	Specificity (%)
ONSD	0.715 (0.630–0.800)	≥4.420	61.4	78.6
ONTA	0.797 (0.723–0.871)	≤175.0	92.9	60.0
Foramen magnum anteroposterior diameter	0.759 (0.679–0.838)	≤38.400	52.9	92.9
Foramen magnum area	0.674 (0.585–0.763)	≤869.554	52.9	77.1
FMI	0.763 (0.684–0.842)	≤1.252	52.9	94.3
aSAS	0.682 (0.594–0.770)	≤6.300	51.4	90.0
Combined multivariable logistic regression model	0.904 (0.852–0.956)	≥0.590	81.4	87.1

Note: ROC curve analysis was performed for radiological parameters that showed significant differences between the IIH and control groups. Optimal cut-off values were determined using the Youden index (J = Sensitivity + Specificity − 1). PPV and NPV are calculated at the Youden index optimal cut-off. FMI ≤ 1.252: PPV = 90.2%, NPV = 66.7%, Youden J = 0.471. aSAS ≤ 6.300 mm: PPV = 83.7%, NPV = 64.9%, Youden J = 0.414. The combined model cut-off of ≥0.590 refers to the predicted probability from the logistic regression equation and is sample-specific. AUC, area under the curve; CI, confidence interval; PPV, positive predictive value; NPV, negative predictive value.

## Data Availability

The data presented in this study are available from the corresponding author upon reasonable request. The data are not publicly available due to privacy and ethical restrictions.

## References

[1] Mollan S.P., Davies B., Silver N.C., Shaw S., Mallucci C.L., Wakerley B.R., Krishnan A., Chavda S.V., Ramalingam S., Edwards J. (2018). Idiopathic intracranial hypertension: Consensus guidelines on management. J. Neurol. Neurosurg. Psychiatry.

[2] Markey K.A., Mollan S.P., Jensen R.H., Sinclair A.J. (2016). Understanding idiopathic intracranial hypertension: Mechanisms, management, and future directions. Lancet Neurol..

[3] Wall M. (2010). Idiopathic intracranial hypertension. Neurol. Clin..

[4] Veiga-Canuto D., Carreres-Polo J. (2020). Role of Imaging in pseudotumor cerebri syndrome. Radiologia.

[5] Bidot S., Bruce B.B., Saindane A.M., Newman N.J., Biousse V. (2015). Asymmetric papilledema in idiopathic intracranial hypertension. J. Neuroophthalmol..

[6] Agid R., Farb R.I., Willinsky R.A., Mikulis D.J., Tomlinson G. (2006). Idiopathic intracranial hypertension: The validity of cross-sectional neuroimaging signs. Neuroradiology.

[7] Enzmann D.R., Pelc N.J. (1992). Brain motion: Measurement with phase-contrast MR imaging. Radiology.

[8] Bhadelia R.A., Bogdan A.R., Wolpert S.M. (1998). Cerebrospinal fluid flow waveforms: Effect of altered cranial venous outflow. A phase-contrast MR flow imaging study. Neuroradiology.

[9] Carlier P., Lantonkpode R., Peltier J., Balédent O., Capel C. (2025). Prognostic value of the preoperative study of cerebrospinal fluid dynamics in Chiari malformations: A pilot study. Acta Neurochir..

[10] Friedman D.I., Liu G.T., Digre K.B. (2013). Revised diagnostic criteria for the pseudotumor cerebri syndrome in adults and children. Neurology.

[11] Farb R.I., Vanek I., Scott J.N., Mikulis D.J., Willinsky R.A., Tomlinson G., terBrugge K.G. (2003). Idiopathic intracranial hypertension: The prevalence and morphology of sinovenous stenosis. Neurology.

[12] Rohr A., Dörner L., Stingele R., Buhl R., Alfke K., Jansen O. (2007). Reversibility of venous sinus obstruction in idiopathic intracranial hypertension. AJNR Am. J. Neuroradiol..

[13] Biousse V., Bruce B.B., Newman N.J. (2012). Update on the pathophysiology and management of idiopathic intracranial hypertension. J. Neurol. Neurosurg. Psychiatry.

[14] Brodsky M.C., Vaphiades M. (1998). Magnetic resonance imaging in pseudotumor cerebri. Ophthalmology.

[15] Degnan A.J., Levy L.M. (2011). Pseudotumor cerebri: Brief review of clinical syndrome and imaging findings. AJNR Am. J. Neuroradiol..

[16] Maralani P.J., Hassanlou M., Torres C., Chakraborty S., Kingstone M., Patel V., Zackon D., Bussière M. (2012). Accuracy of brain imaging in the diagnosis of idiopathic intracranial hypertension. Clin. Radiol..

[17] Sarrami A.H., Bass D.I., Rutman A.M., Alexander M.D., Aksakal M., Zhu C., Levitt M.R., Mossa-Basha M. (2022). Idiopathic intracranial hypertension imaging approaches and the implications in patient management. Br. J. Radiol..

[18] Barkatullah A.F., Leishangthem L., Moss H.E. (2021). MRI findings as markers of idiopathic intracranial hypertension. Curr. Opin. Neurol..

[19] Hoffmann J., Huppertz H.J., Schmidt C., Kunte H., Harms L., Klingebiel R., Wiener E. (2013). Morphometric and volumetric MRI changes in idiopathic intracranial hypertension. Cephalalgia.

[20] Staartjes V.E., Öhlén E., Lilja A., Edström E., Elmi-Terander A. (2025). Editorial: Cerebrospinal fluid flow imaging in Chiari Malformation type 1. Acta Neurochir..

[21] Mugge L., Dang D., Curry B., Whiting R., Crimmins M. (2022). Superior Ophthalmic Vein Flow Patterns as a Marker of Venous Sinus Stenosis and Hypertension in Idiopathic Intracranial Hypertension: A Case of Emergent Transverse Sinus Stenting as Treatment of Fulminant Idiopathic Intracranial Hypertension. World Neurosurg..

[22] Hoffmann J., Mollan S.P., Paemeleire K., Lampl C., Jensen R.H., Sinclair A.J. (2018). European headache federation guideline on idiopathic intracranial hypertension. J. Headache Pain.

[23] Mitchell J.L., Mollan S.P., Vijay V., Sinclair A.J. (2019). Novel advances in monitoring and therapeutic approaches in idiopathic intracranial hypertension. Curr. Opin. Neurol..

[24] Romozzi M., Zeoli F., Martinelli R., Tosto F., Funcis A., Garignano G., Chiloiro S., Di Nardo L., Vollono C., Olivi A. (2025). Cerebrospinal fluid and serum biomarkers in idiopathic intracranial hypertension: A systematic review. Headache.

[25] Yang Y., Ouyang Q., Sun Y., Liang A., Wang J., Wang K., Yuan Y., Liu Z., Huang J., Zhang Z. (2026). Therapeutic Hypothermia Alleviates Hydrocephalus and Neurological Dysfunction Post Intraventricular Hemorrhage by Enhancing Drainage of Glymphatic-Meningeal Lymphatic-Deep Cervical Lymphatic System. CNS Neurosci. Ther..

[26] Tan C., Wang X., Wang Y., Wang C., Tang Z., Zhang Z., Liu J., Xiao G. (2021). The Pathogenesis Based on the Glymphatic System, Diagnosis, and Treatment of Idiopathic Normal Pressure Hydrocephalus. Clin. Interv. Aging.

